# The impact of social determinants of health on chronic kidney disease risk: evidence from the CHARLS study

**DOI:** 10.3389/fpubh.2025.1532372

**Published:** 2025-03-04

**Authors:** Kehua Li, Xue Chen, Lang Chen, Yaorong Liu, Jian Huang, Peixia Li, Dianyin Liang, Jingyu Chen

**Affiliations:** ^1^Department of Physiology and Pathophysiology, Yulin Campus of Guangxi Medical University, Yulin, China; ^2^Department of Basic Medical Experiment Teaching Center, Yulin Campus of Guangxi Medical University, Yulin, China; ^3^Department of Stomatology, People's Hospital of Luchuan, Yulin, China; ^4^Department of Hepatobiliary and Gastrointestinal Surgery, People's Hospital of Beiliu, Yulin, China; ^5^Department of Gynecology, People's Hospital of Beiliu, Yulin, China; ^6^Department of Endocrinology, The Sixth Affiliated Hospital of Guangxi Medical University, Yulin, China; ^7^Department of Public Health, School of Medicine, Guangxi University of Science and Technology, Liuzhou, China; ^8^Department of Endocrine and Metabolic Nephrology, The Affiliated Tumor Hospital of Guangxi Medical University, Nanning, China

**Keywords:** social determinants of health, chronic kidney disease, CHARLS, Chinese, older people

## Abstract

**Background:**

Empirical evidence regarding the relationship between social determinants of health (SDH) and renal outcomes remains limited. Consequently, the objective of this study was to investigate the potential association between SDH and the development of chronic kidney disease (CKD) across various levels.

**Methods:**

Data were sourced from the 2011 China Health and Retirement Longitudinal Study (CHARLS), which included 6,290 Chinese participants aged 40 years and older. Among these participants, 4,115 underwent a follow-up assessment in the 2015 survey. The primary outcome measure was the incidence of CKD, operationally defined as a reduction in estimated glomerular filtration rate to <60 ml/min/1.73 m^2^. To analyze the association between varying levels of SDH and renal outcomes, a Cox proportional hazards regression model was employed.

**Results:**

The findings indicate that, in comparison to individuals with a pension, higher education, and no need for family support, the risk of developing CKD increased by 43, 49, and 52%, respectively. Furthermore, the combination of requiring family support, being unmarried, and lacking medical insurance was associated with an elevated incidence of CKD. Utilizing the counting model of adverse SDH indicators, it was observed that when the number of adverse SDH was equal to or greater than four, there was a significant increase in the risk of CKD. The incidence density of CKD was found to rise in correlation with the severity of adverse SDH, with the incidence density in the adverse SDH group being 0.06 per person-year higher than that in the favorable SDH group. After adjusting for multiple variables, the hazard ratio (HR) for incident CKD was 2.47 [95% confidence interval (CI): 1.46–4.16] in the adverse SDH group compared to the favorable SDH group, a finding that persisted across various subgroups.

**Conclusion:**

Research indicates that financial support, pensions, education, marital status, and health insurance significantly impact CKD risk. Higher income, pension coverage, education, marital stability, and insurance lower this risk. Evaluating adverse SDH indicators helps assess individual SDH levels and CKD risk, with four or more indicators suggesting high risk. Therefore, adverse SDH measures can predict CKD.

## 1 Introduction

CKD encompasses a range of renal disorders marked by enduring structural and functional impairments persisting over several months (1). Over recent decades, CKD has emerged as a significant global public health challenge, associated with substantial management costs and suboptimal treatment outcomes. The international consensus underscores that early detection is a crucial strategy for preventing nephropathy, its progression, and associated complications. However, numerous studies indicate that public awareness of nephropathy remains insufficient, CKD has ascended to become the seventh leading risk factor for mortality globally (2). The prevalence of CKD in China is ~10.8%, with an estimated 119.5 million individuals affected (3). This suggests that, notwithstanding the availability of certain medical resources, there remains a critical need to enhance awareness and management of CKD to mitigate its impact on public health. Consequently, it is imperative to formulate effective prevention policies, identify and treat high-risk populations, and prioritize early detection and intervention strategies for CKD (2, 4).

The World Health Organization (WHO) introduced the concept of the SDH, which it defines as “the social and environmental characteristics affecting individuals from birth through growth, life, work, and aging” (5). This concept highlights the fundamental framework of social stratification and its influence on individual living and working conditions, which subsequently affect health outcomes. The examination of social determinants is instrumental in identifying the adverse social and environmental factors associated with CKD. Research indicates that a low socioeconomic status is linked to an increased incidence of CKD (6). A study examining racial disparities among CKD patients in the United States supports this finding, revealing that Black individuals with low socioeconomic status frequently experience a higher incidence of CKD compared to their White counterparts (7). Considering the variations in social policy measures across different countries and the ethnic distinctions between Chinese and Western populations, the impact of SDH on the incidence of CKD in China remains uncertain.

Research indicates that social determinants impact individual health behaviors and indirectly influence CKD risk by affecting healthcare access and lifestyle choices (8). For instance, individuals with lower educational attainment frequently lack access to health insurance and are less likely to engage in healthy lifestyle practices, potentially resulting in a higher prevalence of CKD (9). Moreover, the impact of the social environment is significant; patients residing in communities characterized by lower socioeconomic status often experience higher incidence rates and less favorable treatment outcomes for CKD (10). Consequently, comprehending the role of social determinants in the onset and progression of CKD is essential for formulating effective public health policies and interventions. Nevertheless, none of the aforementioned studies have investigated the relationship between SDH and CKD within a larger or national population. Numerous studies have predominantly concentrated on the effects of individual SDH indicators on CKD (11, 12). While some researchers have utilized the CHARLS dataset to examine the risk of falls in CKD patients and the influence of circadian rhythm synthesis on CKD risk (13, 14), there remains a lack of research specifically addressing the relationship between SDH factors and CKD.

This study seeks to analyze how SDH factors influence CKD outcomes in China using the CHARLS dataset. CHARLS is a national panel survey of China's middle-aged and older adults, covering over 337 million people. It includes social determinants of health indicators like socioeconomic status, education, healthcare usage, and health spending (15). This initiative is anticipated to enhance patient health outcomes while simultaneously advancing health equity on a larger scale (16).

## 2 Methods

### 2.1 Participants

We utilized data from two waves of CHARLS database. CHARLS is a nationally representative, population-based longitudinal health survey administered by the National School of Development at Peking University, targeting individuals aged 45 years and older. The CHARLS conducted a comprehensive national baseline survey in 2011, encompassing 28 provinces, 150 counties/districts, and 450 villages/urban communities. A baseline survey was conducted in 2011 and follow-up surveys in 2013, 2015, and 2018 were conducted (17). A multi-stage probability sampling strategy was employed to select respondents. Due to the availability of individual blood data solely within the public database for the 2011 baseline survey and the 2015 survey, this study was restricted to data spanning from 2011 to 2015.

**Figure 1:**
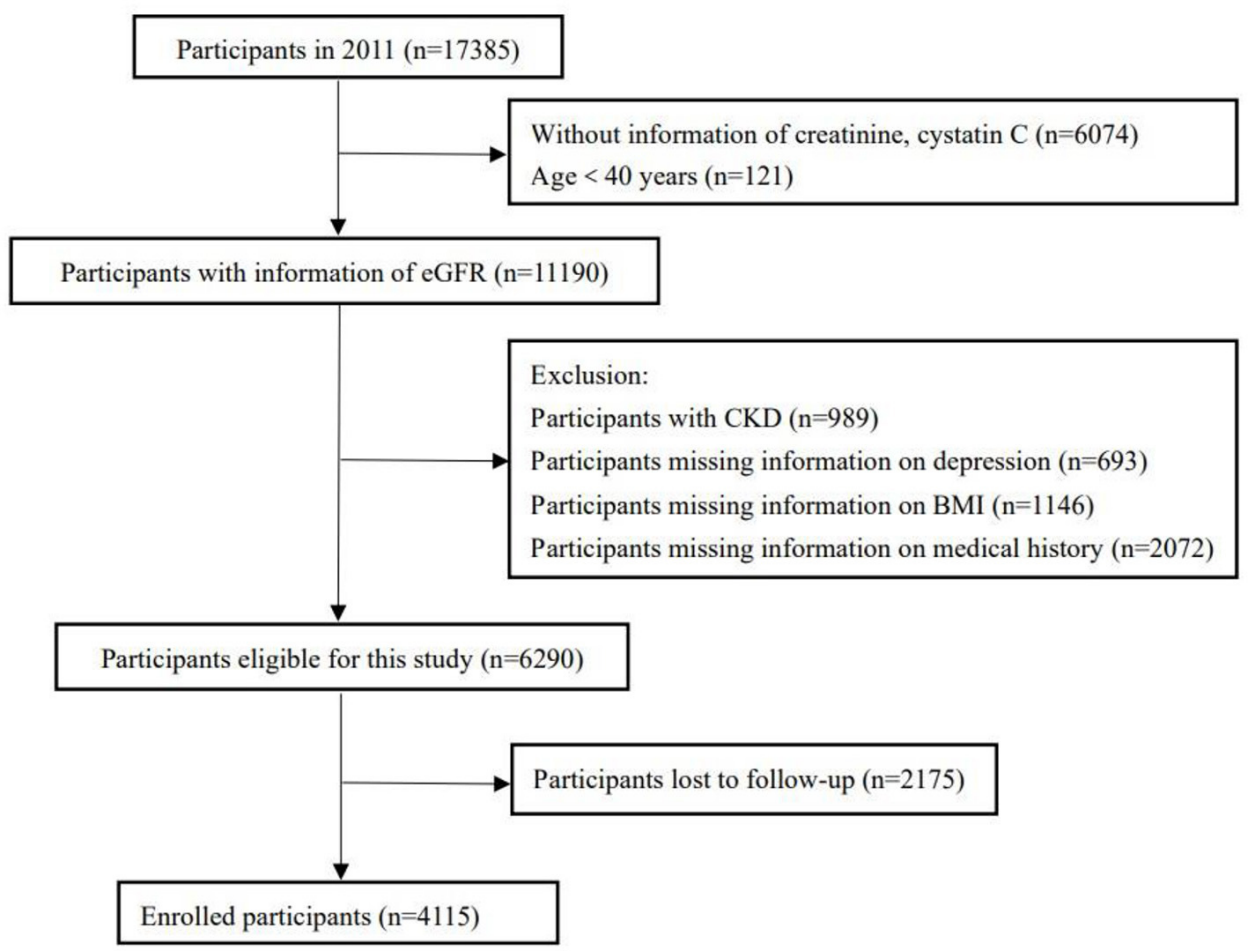
Flow chart for participants selection and follow-up.

A longitudinal cohort study was implemented utilizing baseline data from the CHARLS in 2011 and follow-up data from 2015. Initially, the survey in 2011 included 17,385 participants. After excluding individuals lacking essential information, such as renal function data, and those under 40 years of age, 11,190 participants satisfied the inclusion criteria. Participants with an estimated glomerular filtration rate (eGFR) below 60 ml/min/1.73 m^2^ in the 2011 dataset (*n* = 989) and those missing medical history, body mass index (BMI), or depression scale scores (*n* = 3,911) were also excluded. Consequently, 6,290 participants were monitored over a four-year period, with further exclusions for those lost to follow-up (*n* = 2,175). Ultimately, the final sample for the longitudinal cohort study comprised 4,115 participants, as illustrated in Figure 1.

The Biomedical Ethics Review Committee of Peking University granted ethical approval for data collection in the CHARLS database (Approval No. IRB00001052-11015). All participants in the CHARLS database provided informed consent.

### 2.2 Data sources

Baseline data encompassing individual characteristics, sociodemographic information, physical measurements, and both physical and mental health status were collected through a structured questionnaire. The outcome measures for CKD were derived from summary data collected during the follow-up period in 2015. The exposure variables, encompassing demographic and clinical data—specifically age, BMI, sex, sleep duration, marital status, residence, educational attainment, smoking status, alcohol consumption, and medical history (including hypertension, dyslipidemia, diabetes, heart disease, kidney disease, stroke, cancer, and disability)—were derived from baseline data collected in 2011. Additionally, venous blood samples were obtained from fasting participants by trained nursing staff for the assessment of biochemical indicators, including serum creatinine (Scr), serum cystatin C (SCysC), and urea nitrogen.

The educational attainment was determined through the inquiry, “What is the highest level of education you have currently achieved?” The responses were categorized into two distinct groups: elementary school or below, and middle school or above. Marital status was classified into two categories: married and unmarried, with the latter encompassing divorced, widowed, and never-married individuals. Non-smokers were identified as individuals who had never engaged in cigarette smoking, whereas smokers included those who had either previously smoked or were actively smoking cigarettes. An individual was classified as an alcohol consumer if they currently consumed alcohol more than once per month or had done so in the past. Medical history was collected by inquiring whether participants had ever been diagnosed by a physician with any specified chronic conditions. Sleep duration was evaluated through a self-reported question, which asked respondents, “In the past month, how many hours of actual sleep did you obtain at night on average? This may be less than the total hours spent in bed.”

The CES-D, a tool used to measure the intensity of depressive symptoms, was utilized in CHARLS (18). It is a validated instrument for assessing depression among the Chinese population. The CES-D includes 10 questions, each scored on a 4-point Likert scale, where participants report how often they experienced depressive symptoms in the previous week. Scores range from 0 to 30, with scores of 10 or above suggesting depression (19).

### 2.3 Assessment of the SDH

The WHO overview of SDHs was referenced, and the five components of the “Healthy People 2030” organizational framework were adopted. SDHs were chosen as the domain of focus, encompassing the following five domains: economic stability, education, social/community environment, healthcare, and neighborhood/built environment.

#### 2.3.1 A section

Income and occupation were categorized under economic stability. Income instability is characterized by the lack of pensions and the necessity for financial support from family members, such as children. Retirement and unemployment were identified as adverse occupational factors. Within the educational dimension, low educational attainment, defined as having completed only primary school or less, was considered a disadvantage. In the social/community environment dimension, poor marital status, encompassing individuals who were widowed, divorced, separated, or never married, was identified as a disadvantage. In the healthcare dimension, the absence of health insurance was regarded as a disadvantage. The neighborhood/built environment dimension includes factors such as regional gross domestic product (GDP) per capita, air quality, and place of residence. GDP data were sourced from the statistical yearbook, and a regional GDP per capita lower than the national GDP per capita for the same year was considered a disadvantage. According to the secondary standard for ambient PM_2.5_ outlined in the “Ambient Air Quality Standard” (GB 3095-2012), a PM_2.5_ concentration exceeding 75 μg/m^3^ was classified as high exposure and deemed an adverse factor (20). Residing in a rural area has been identified as a disadvantageous residential factor.

#### 2.3.2 B section

In the economic stability dimension, factors such as possessing a pension, the absence of a need for familial support, and maintaining employment status were identified as advantageous. Within the educational dimension, attaining an education level beyond primary school was deemed beneficial. In the social and community environment dimension, marital status emerged as a favorable factor. Regarding the healthcare dimension, the possession of medical insurance was considered advantageous. Finally, within the neighborhood and built environment dimension, a regional GDP per capita exceeding the national average for the same year, a PM_2.5_ concentration of 75 μg/m^3^ or lower, and residing in urban areas were recognized as favorable factors.

### 2.4 Outcome

We employed the Chronic Kidney Disease Epidemiology Collaboration (CKD-EPI Scr-SCysC combined formula) creatinine-cystatin C equation (21) to determine the estimated glomerular filtration rate (eGFR), as presented below:


$$\begin{array}{l} \text{eGFR }(\text{mL/min/1}.{\text{73 m}}^{2})={\text{a}}^{*}{\left(\text{Scr/b}\right)}^{\wedge }{\text{c}}^{*}{\left(\text{SCysC/0}.\text{8}\right)}^{\wedge }{\text{d}}^{*}\\ \;{\left(\text{0}.\text{995}\right)}^{\wedge }\text{age}. \end{array}$$

The following values were reported: (1) a-values were 130 for women and 135 for men; (2) b-values were 0.7 for women and 0.9 for men; (3) c-values for females with Scr ≤ 0.7 mg/dL were −0.248, and −0.601 for those with Scr > 0.7 mg/dL. For males, the c-values were −0.207 with Scr ≤ 0.9 mg/dL and −0.601 with Scr > 0.9 mg/dL; (4) d-values were −0.375 for SCysC ≤ 0.8 mg/L and −0.711 for SCysC > 0.8 mg/L.

The CKD-EPI equation that integrates both Scr and SCysC demonstrates superior precision and accuracy in estimating the GFR compared to equations utilizing either Scr or SCysC independently. Consequently, the CKD-EPI combined equation significantly decreases the likelihood of incorrectly diagnosing individuals with CKD when they do not, in fact, have the condition (21). In the CHARLS study, the Scr was measured using a picric acid assay, while Scys was assessed through immune turbidimetry (22). The primary outcome of the study was the development of CKD, defined as a reduction in the eGFR to below 60 ml/min/1.73 m^2^ (23).

### 2.5 Statistical analysis

Measurement data with a normal distribution were summarized as mean ± standard deviation, and compared using independent sample *t*-tests. For skewed data, the median and interquartile range were used, with the Mann-Whitney *U*-test for comparisons. Enumeration data were presented as case numbers and ratios, with chi-square tests for group comparisons.

The Cox proportional hazards regression model, along with the stepwise regression method (also known as the stepwise screening method), was employed to elucidate the associations between SDH and renal outcomes. Two distinct models were proposed: Model 1, referred to as the unadjusted or crude model, did not account for any covariates; whereas Model 2 was adjusted for a range of variables, including gender, BMI, smoking status, alcohol consumption, hypertension, diabetes, depression, dyslipidemia, cardiovascular disease, stroke, and sleep duration. Initially, we conducted a univariate difference analysis. Subsequently, in conjunction with existing literature (13), the variables identified as statistically significant in the univariate analysis were incorporated as covariates in the Cox regression model to assess the association between SDH and CKD. In this study, a comprehensive evaluation of SDH was developed using the index counting method. Initially, SDH scores associated with the onset of CKD were derived from the coefficients of each SDH index within a stepwise regression model. Subsequently, participants were categorized into unfavorable and favorable SDH groups based on the dose-response relationship between SDH and CKD risk. The HR and 95% CI for varying SDH levels and CKD incidence were then estimated.

Statistical analyses were performed using SPSS software (version 26), and Origin software (version 9.8), with all *p*-values being two-sided and a threshold of *P* < 0.05 considered statistically significant.

## 3 Results

### 3.1 Baseline characteristics

This cohort study comprised 4,115 eligible participants. During the follow-up period, 195 new cases of CKD were identified, representing 4.74% of the study population. The baseline characteristics of the participants are detailed in Table 1. The participants had a mean age of 58.36 ± 8.68 years, with 42.45% being male. The average sleep duration among participants was 6.36 ± 1.88 h. The mean eGFR was 89.31 ml/min/1.73 m^2^. Notably, the incidence of CKD was higher in male participants compared to female participants. The cohort with CKD was distinguished by an older age demographic, a higher prevalence of unmarried individuals, reduced sleep duration, lower educational attainment, and an increased proportion of cigarette smokers in comparison to the non-CKD cohort. Additionally, the CKD group exhibited a lower incidence of medical insurance and pension coverage and demonstrated greater reliance on familial financial support, such as alimony for the older adults. Furthermore, this group showed elevated rates of comorbid conditions, including hypertension, diabetes mellitus, dyslipidemia, disability, and stroke (refer to Table 1).

**Table 1:** Characteristics of baseline population.

**Variables**	**Total (*n* = 4,115)**	**CKD group (*n* = 195)**	**Non-CKD group (*n* = 3,920)**	***P***
Age, years	58.36 ± 8.68	65.04 ± 8.19	58.02 ± 8.57	<0.001
BMI, Kg/m^2^	23.67 ± 3.84	23.91 ± 4.56	23.65 ± 3.80	0.368
Sleep duration, hours	6.36 ± 1.88	5.92 ± 2.07	6.39 ± 1.87	0.001
eGFR, ml/min/1.73 m^2^	89.31 (78.20, 101.30)	52.34 (50.34, 57.38)	91.13 (80.13, 101.79)	<0.001
**Gender**, ***n*** **(%)**				<0.001
Male	1,747 (42.45)	112 (57.44)	1,635 (41.71)	
Female	2,368 (57.55)	83 (42.56)	2,285 (58.29)	
**SDH**, ***n*** **(%)**				
Family support	1,470 (35.72)	93 (47.69)	1,377 (35.13)	<0.001
Retired	412 (10.01)	26 (13.33)	386 (9.85)	0.113
Non-pensioner	3,166 (76.94)	127 (65.13)	3,039 (77.53)	<0.001
Elementary school or below	2,929 (71.18)	152 (77.95)	2,777 (70.84)	0.032
Unmarried or others	451 (10.96)	39 (20.00)	412 (10.51)	<0.001
No health insurance	207 (5.03)	16 (8.21)	191 (4.87)	0.038
Rural	2,757 (67.00)	121 (62.05)	2,636 (67.24)	0.132
High PM_2.5_	593 (14.41)	24 (12.31)	569 (14.52)	0.392
Low urban per capita GDP	2,564 (62.31)	122 (62.56)	2,442 (62.30)	0.940
**Smoking**, ***n*** **(%)**				0.008
Smoker	1,157 (28.12)	71 (36.41)	1,086 (27.70)	
Non-smoker	2,958 (71.88)	124 (63.59)	2,834 (72.30)	
**Drinking**, ***n*** **(%)**				0.334
Drinker	1,285 (31.23)	67 (34.36)	1,218 (31.07)	
Non-drinker	2,830 (68.77)	128 (65.64)	2,702 (68.93)	
**Medical history**, ***n*** **(%)**				
Hypertension	1,032 (25.08)	81 (41.54)	951 (24.26)	<0.001
Diabetes	250 (6.08)	20 (10.26)	230 (5.87)	0.012
Heart disease	506 (12.30)	32 (16.41)	474 (12.09)	0.073
Stroke	76 (1.85)	9 (4.62)	67 (1.71)	0.003
Dyslipidemia	417 (10.13)	32 (16.41)	385 (9.82)	0.003
Disability	654 (15.89)	43 (22.05)	611 (15.59)	0.016
Depression	1,594 (38.74)	74 (37.95)	1,520 (38.78)	0.817

### 3.2 The SDH associated with the incidence of CKD

**Figure 2:**
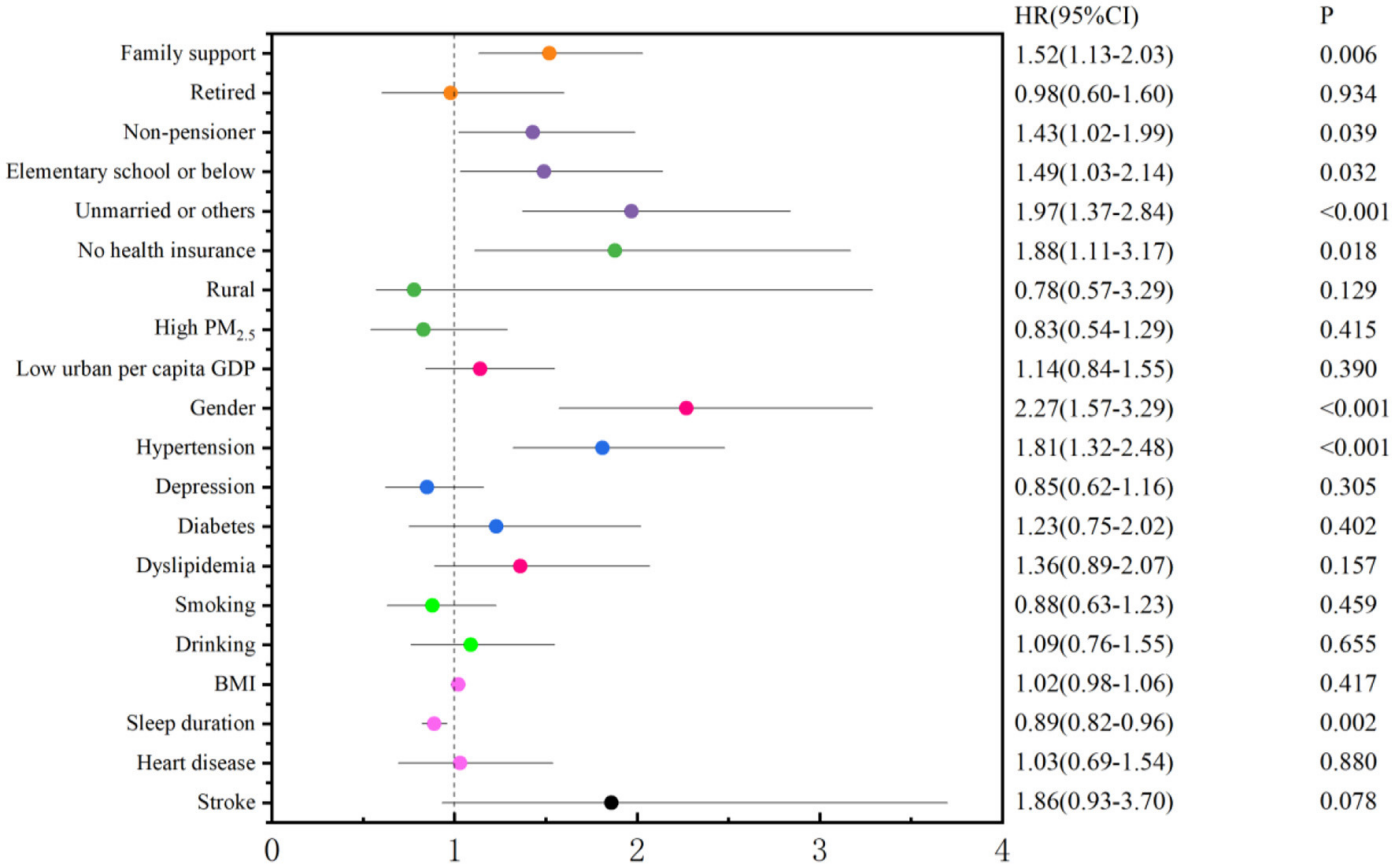
Forest plot of the SDHs of CKD incidence.

The SDH indicators were incorporated into the regression model to examine their association with the onset of CKD. The findings from the stepwise regression analysis indicated that the model, with CKD as the dependent variable, identified five significant SDH indicators: the need for family support, lack of pension, low educational attainment, unmarried status, and absence of medical insurance. These results demonstrated that the need for family support, absence of a pension, low education level, unmarried status, and lack of medical insurance were adverse SDH factors significantly correlated with the incidence of CKD (Figure 2).

**Figure 3:**
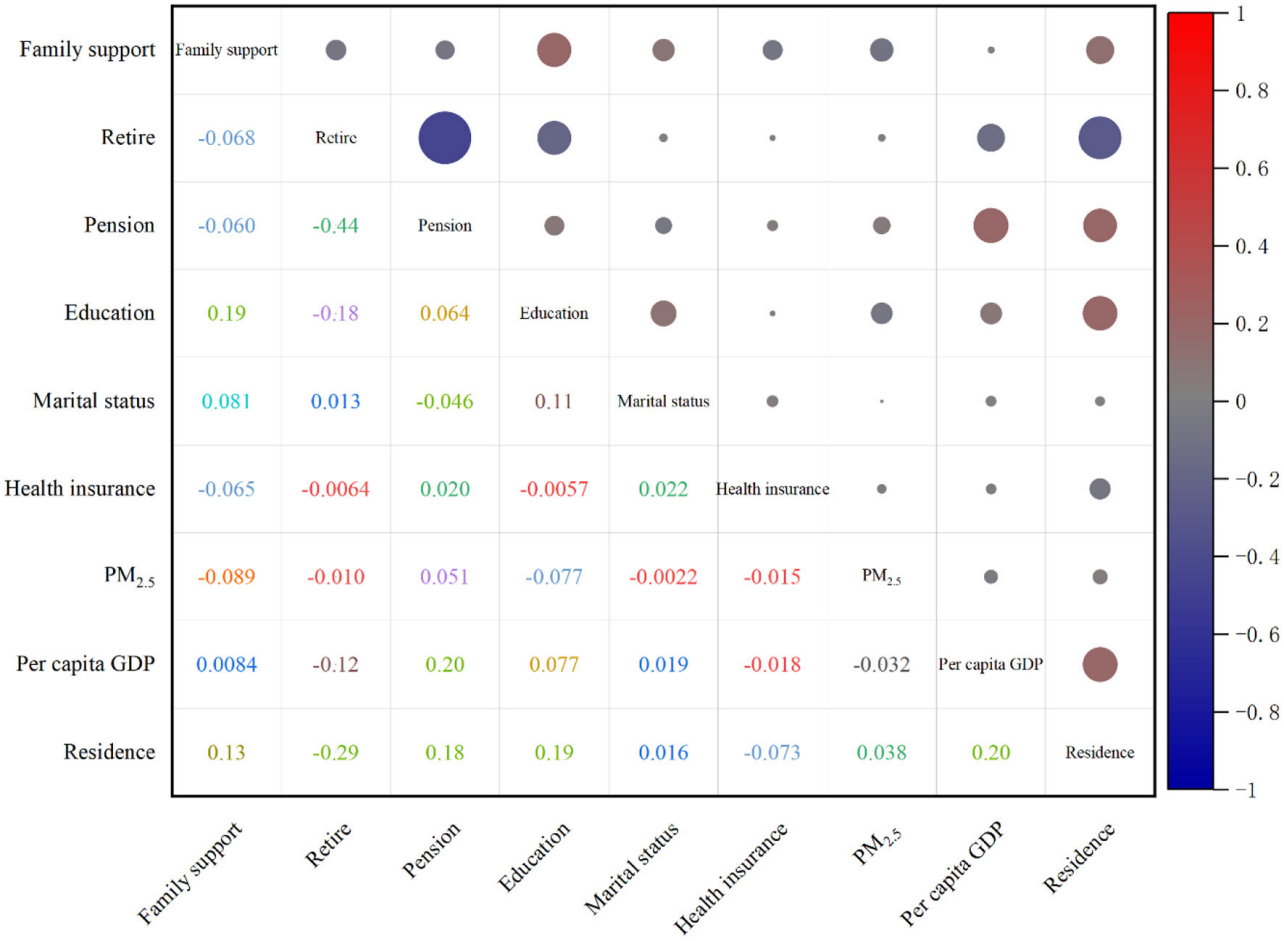
SDH indicator correlation coefficient matrix (the upper right corner matrix represents the correlation size, the lower left corner matrix is the correlation coefficient, and the right bar is the correlation coefficient contrast color).

The incidence density of CKD was observed to be 19.70, 18.23, and 9.15 per person-year among individuals without a pension, with a low level of education, and those requiring family support, respectively. The risk of developing CKD increased by 43, 49, and 52% for individuals lacking a pension, possessing a low educational level, and needing family support, in comparison to those with a pension, higher education, and no need for family support, respectively. The correlations among SDH indicators are presented in Figure 3, with the strongest correlation observed between pension status and retirement, exhibiting a correlation coefficient of −0.44. The correlation coefficients between retirement and residence, pension and local GDP per capita, and pension and local GDP per capita were −0.29, 0.24, and −0.23, respectively, as presented in Table 2 and illustrated in Figure 3.

**Table 2:** Correlation between each SDH indicator and CKD in the stepwise regression model.

**SDH**	**Number of cases**	Incidence density^*^	**Model 1**		**Model 2**	
			**HR (95%CI)**	***P***	**HR (95%CI)**	***P***
Family support	1,470	9.15	1.53 (1.14–2.06)	0.004	1.52 (1.13–2.03)	0.006
Retired	412	2.56	1.07 (0.66–1.74)	0.783	0.98 (0.60–1.60)	0.934
Non-pensioner	3,166	19.7	1.67 (1.19–2.32)	0.003	1.43 (1.02–1.99)	0.039
Elementary school or below	2,929	18.23	1.35 (0.94–1.94)	0.099	1.49 (1.03–2.14)	0.032
Unmarried or others	451	2.81	1.75 (1.23–2.51)	0.002	1.97 (1.37–2.84)	<0.001
No health insurance	207	1.29	1.73 (1.03–2.90)	0.038	1.88 (1.11–3.17)	0.018
Rural	2,757	17.16	0.78 (0.57–1.08)	0.133	0.78 (0.57–3.29)	0.129
High PM_2.5_	593	3.69	0.95 (0.62–1.46)	0.820	0.83 (0.54–1.29)	0.415
Low urban per capita GDP	2,564	15.96	1.42 (0.85–1.54)	0.389	1.14 (0.84–1.55)	0.390

Model 1: was unadjusted.

Model 2: was adjusted for gender, BMI, smoking, drinking, hypertension, diabetes, depression, dyslipidemia, heart disease, stroke, sleep duration.

* Per person-year.

### 3.3 Combined impact of SDH on the incidence of CKD

Significant differences were observed in the incidence of CKD among individuals requiring family support, those who were unmarried, and those lacking medical insurance. Consequently, we conducted a further investigation into the association between the need for family support, unmarried status, and absence of medical insurance with the incidence of CKD. The results indicated that, relative to subjects requiring family support and lacking medical insurance, those who did not require family support and lacked medical insurance, as well as those who neither required family support nor lacked medical insurance, exhibited a 75, 78, and 84% reduced risk of CKD, respectively. Furthermore, compared to unmarried and uninsured individuals, married and insured subjects demonstrated a 72% lower risk of CKD. In comparison to individuals who were unmarried and required family support, those who were married and did not require such support exhibited a 65% reduced risk of CKD (refer to Table 3).

**Table 3:** Combined impact of SDH on the incidence of CKD.

**SDH**	**Number of cases**	Incidence density^*^	**HR (95%CI)**	***P***
**Family support and health insurance**				
FS+/HI−	46	0.29	Reference	
FS+/HI+	1,424	8.86	0.25 (0.12–0.51)	<0.001
FS−/HI−	161	1	0.22 (0.81–0.58)	0.002
FS/HI+	2,484	15.46	0.16 (0.08–0.36)	<0.001
**Marital status and health insurance**				
MS−/HI−	29	0.18	Reference	
MS−/HI+	422	2.62	0.59 (0.21–1.56)	0.326
MS+/HI−	178	1.11	0.50 (0.16–1.56)	0.231
MS+/HI+	3,486	21.69	0.28 (0.10–0.75)	0.012
**Marital status and family support**				
MS−/FS+	211	1.31	Reference	
MS−/FS−	240	1.49	0.92 (0.49–1.73)	0.787
MS+/FS+	1,259	7.83	0.60 (0.35–1.01)	0.054
MS+/FS−	2,405	14.97	0.35 (0.21–0.59)	<0.001

Cox proportional hazards regression model: was adjusted for gender, BMI, smoking, drinking, hypertension, diabetes, depression, dyslipidemia, heart disease, stroke, sleep duration.

* Per person-year.

### 3.4 Prediction of CKD incidence by comprehensive evaluation indicators of SDH

A counting model of adverse SDH indicators was employed to quantify the cumulative number of adverse SDH indicators associated with the onset of CKD, thereby reflecting the individual's comprehensive exposure to SDH. The risk of CKD increased significantly when the number of adverse SDH indicators was two or more, continued to rise steadily with two or more indicators, and escalated substantially when the number of adverse SDH indicators reached four or more. In the analysis of adverse SDH, groups exhibiting similar effect sizes were consolidated. The adverse SDH associated with CKD incidence were categorized into favorable SDH (fewer than four adverse SDH) and unfavorable SDH (four or more adverse SDH) (refer to Table 4).

**Table 4:** Prediction of CKD incidence by comprehensive evaluation indicators of SDH.

**Number of adverse SDHs**	**Number of cases**	Incidence density^*^	**HR (95%CI)**	***P***
0	213	1.32	Reference	
1	982	6.11	0.95 (0.47–1.92)	0.894
2	1,695	10.55	1.13 (0.58–2.21)	0.729
3	1,054	6.56	1.64 (0.83–3.23)	0.157
4	166	1.03	2.73 (1.19–6.29)	0.018
5	5	0.03	16.67 (3.55–78.35)	<0.001

Cox proportional hazards regression model: was adjusted for gender, BMI, smoking, drinking, hypertension, diabetes, depression, dyslipidemia, heart disease, stroke, sleep duration;

* Per person-year.

### 3.5 Association between the level of SDH and the incidence of CKD and subgroup analysis

The incidence density of CKD was observed to increase with the severity of adverse SDH, with the incidence density in the unfavorable SDH group being 0.06 per person-year higher than that in the favorable SDH group. After adjusting for multiple variables, the HR for CKD incidence in the unfavorable SDH group, compared to the favorable SDH group, was 2.47 (95% confidence interval: 1.46–4.16). In individuals under the age of 60, the incidence of CKD was higher in the group with unfavorable SDH compared to those with favorable SDH. Unfavorable SDH was associated with a 2.85-fold increased risk of CKD in men (95% CI: 1.53–5.31), but this association was not observed in women. Among individuals with a sleep duration of 6 h or more and those without depression, the risk of CKD was higher in the unfavorable SDH group compared to the favorable SDH group, with hazard ratios of 3.07 (95% CI: 1.47–6.41) and 3.61 (95% CI: 1.87–9.97), respectively. Furthermore, the likelihood of developing CKD was greater in the unfavorable SDH group compared to the favorable SDH group, across both hypertensive and non-hypertensive populations, with no observed heterogeneity in the association between these groups and CKD incidence (refer to Table 5).

**Table 5:** Association between the level of SDH and the incidence of CKD and subgroup analysis.

**Subgroup**	**Number of cases**	Incidence density^*^	**Model 1**		**Model 2**	
			**HR (95%CI)**	***P***	**HR (95%CI)**	***P***
Family support	171	1.06	2.06 (1.24–3.44)	0.006	2.47 (1.46–4.16)	0.001
**Age, years**						
<60	51	0.32	2.80 (0.87–8.99)	0.083	3.41 (1.04–11.17)	0.043
≥60	120	0.75	1.37 (0.78–2.42)	0.278	1.77 (0.98–3.18)	0.055
**Gender**						
Male	42	0.26	1.50 (0.55–4.08)	0.423	1.73 (0.63–4.75)	0.287
Female	129	0.80	2.93 (1.59–5.41)	0.001	2.85 (1.53–5.31)	0.001
**Sleep duration, hours**						
<6	79	0.49	1.67 (0.80–3.47)	0.170	1.84 (0.87–3.87)	0.111
≥6	92	0.57	2.23 (1.09–4.57)	0.029	3.07 (1.47–6.41)	0.003
**Depression**						
Yes	94	0.58	1.41 (0.61–3.24)	0.422	1.56 (0.67–3.66)	0.305
No	77	0.48	2.86 (1.50–5.46)	0.001	3.61 (1.87–9.97)	<0.001
**Hypertension**						
Yes	42	0.26	2.23 (1.03–4.84)	0.043	2.86 (1.28–6.36)	0.010
No	129	0.8	1.96 (0.99–3.88)	0.052	2.14 (1.07–4.28)	0.032

Model 1: was unadjusted.

Model 2: was adjusted for gender, BMI, smoking, drinking, hypertension, diabetes, depression, dyslipidemia, heart disease, stroke, sleep duration.

* Per person-year.

## 4 Discussion

The global prevalence of CKD is on the rise. Data from the Global Kidney Health Atlas by the International Society of Nephrology indicates that the median prevalence of CKD in Latin America stands at 10.2% (24). Worldwide, the prevalence of CKD across all age groups has surged by 29.3%, accompanied by a 41.5% increase in the global mortality rate associated with the disease (25). This retrospective cohort study examined the relationship between adverse SDH and CKD incidence, identifying the most impactful SDH indicators and assessing the combined effect of various indicators on CKD incidence. The comprehensive evaluation index model of SDH, which demonstrated a strong predictive capability for CKD, was employed to further examine the risk of CKD across varying levels of SDH. The analysis revealed that suboptimal SDH significantly elevated the risk of CKD. This study contributes to the understanding of social factors influencing CKD risk within the Chinese population and suggests that enhancing SDH could be advantageous in mitigating CKD risk.

In alignment with previous research, the selected SDH indicators were identified as significant contributors to the increased incidence of CKD (7, 8). Previous research has demonstrated a significant correlation between low income and low educational attainment with a heightened incidence of CKD (26). This study further identifies the need for financial support from family members and the absence of a pension as SDH linked to the onset of CKD. Potential explanations for these findings include the reliance of older adults on familial financial support and the lack of a pension, suggesting an absence of stable economic resources or employment benefits. This economic instability may influence their consumption patterns and willingness or ability to seek medical treatment.

Furthermore, this study identified the absence of medical insurance as a SDH that elevates the risk of CKD. The majority of respondents reside in rural areas, where the economic burden of farmers' medical insurance is minimal. However, without medical insurance, families experience financial discomfort, suggesting that individuals facing economic and social challenges are at an increased risk of developing CKD (27). Notably, research conducted on European and American populations has not focused on comparing the evaluative impacts of different quantitative methods on SDH indicators. Instead, these studies have emphasized factors such as race and social isolation, including whether individuals receive care during illness (28, 29). Consequently, the influence of marital status on the incidence of CKD was incorporated into this study. The findings indicate that being unmarried significantly elevates the risk of CKD, potentially due to psychological factors like social isolation. This warrants further investigation in future research. This study included air pollution indicators based on the characteristics of high PM_2.5_ exposure in China, and previous research evidence has also shown that PM2.5 is associated with the incidence of CKD (30, 31). However, SDH indicators related to the incidence of CKD did not include high PM2.5 exposure in this study, possibly due to the inconsistent definition of high PM_2.5_ exposure. In addition, other social factors were included in this study to exclude the potential confounding effect. Furthermore, this study did not establish an association between GDP per capita and residence with the incidence of CKD, indicating the need for further evidence to substantiate these findings.

Among the adverse SDH related to the incidence of CKD, needing family financial support, being unmarried, and having no medical insurance had a significant impact on the incidence of CKD. Additionally, income level, occupation, medical insurance, and education are critical indicators of SES, which play a significant role in kidney health. Moreover, psychosocial factors, such as social isolation, should not be overlooked in the onset and progression of CKD. Psychosocial factors, including social isolation, play a significant role in the onset and progression of CKD. The analysis of combined variables such as the need for family financial support, marital status, and medical insurance revealed a notable effect modification in the relationship between economic status, marital status, and medical insurance with CKD among older adults. Furthermore, the observed association between variations in SDH indicators and CKD suggests that enhancing income levels, ensuring marital stability, and expanding medical insurance coverage may contribute to improved renal health outcomes. This study can provide some research evidence for exploring the control of adverse SDH indicators related to CKD.

In contrast to earlier studies that examined the relationship between individual SDH indicators and CKD (32), this study offers a more comprehensive and systematic evaluation of the association between overall SDH levels and the incidence of CKD. This research assessed the cumulative impact of SDH on individuals by quantifying adverse SDH indicators. The method of counting adverse SDH indicators is straightforward and user-friendly. International researchers have employed this SDH counting approach to identify individuals at elevated risk for coronary heart disease events (33). Consequently, we incorporated SDH indicators that reflect the characteristics of social factors in China and employed an SDH indicator counting method to include variables related to air quality and local economic conditions. This approach facilitated a comprehensive evaluation of individual SDH. The resulting comprehensive evaluation index may serve as a straightforward method to encapsulate the social factors associated with CKD within the Chinese population.

Through subgroup analysis, it was determined that the incidence of CKD was higher in the unfavorable socioeconomic and SDH group compared to the favorable SDH group across various age cohorts under 60 years. However, the incidence density of CKD was greater among individuals over 60 years of age. Notably, adverse SDH significantly elevated the risk of CKD in men, whereas this association was not statistically significant in women. Furthermore, the risk of CKD was greater in the unfavorable SDH group compared to the favorable SDH group among individuals with a sleep duration of 6 h or more and those without depression. Consequently, it was noteworthy to observe the absence of heterogeneity in the association between “disadvantaged” groups—characterized by older age, sleep deprivation, and depression—and both unfavorable and favorable SDH groups with the incidence of CKD. Furthermore, no heterogeneity was detected in the association with CKD incidence between individuals with and without hypertension. Previous research has similarly investigated the relationship between SDH and cardiovascular disease across various subgroups, indicating that social factors may exhibit stronger associations with mortality risk in younger populations (34). The associations between various social factors and CKD or mortality exhibit inconsistencies across genders and age groups, and there is a paucity of research investigating these intergroup differences. Therefore, more subgroup studies are needed to fully understand the impact of SDH on CKD in different genders and age groups, so as to provide more targeted guidance for different populations.

Previous studies have suggested a potential causal relationship between SDH and the incidence of CKD. This relationship is attributed to the impact of social factors on the resources available to individuals, including knowledge, wealth, prestige, access to medical care, social relationships, environmental conditions, and health-related behaviors and factors (6). Material deprivation, financial instability, inadequate living and working conditions, insufficient social support, and limited access to healthcare services, coupled with adverse psychological factors such as elevated occupational stress, can precipitate allostatic stress responses. These responses encompass the activation of stress hormones (35), inflammation (36), endothelial dysfunction, and metabolic disorders (37). Sustained elevation of allostatic load may exert detrimental effects on various organ systems, with particularly pronounced impacts on the cardiovascular system (38). Research indicates that socioeconomic disadvantage exerts long-term physiological impacts, with early exposure to adverse social factors elevating the risk of renal dysfunction (39).

This study aims to establish a theoretical foundation for addressing health disparities arising from social determinants and for developing pertinent policies. The innovative aspect of this research lies in identifying the primary SDH influencing CKD within the Chinese population and in developing a user-friendly comprehensive evaluation index of SDH. This index can be utilized to predict populations at high risk for CKD. The primary strengths of this study include its foundation on a nationally representative prospective cohort, encompassing a substantial sample size drawn from 15 provinces, along with an extended follow-up period and multiple follow-up surveys. Additionally, the large sample size provided sufficient statistical power to conduct both joint and stratified analyses.

Nevertheless, the study is subject to certain limitations: notably, the total income of participants was not collected at the baseline of the CHARLS cohort, and there was a significant amount of missing data regarding total family income. We utilized data from the initial follow-up to supplement missing information, incorporating variables such as financial support from family members, pensions, retirement, and other related data. Furthermore, while our study encompassed at least one SDH measure across all domains of healthy individuals, certain potentially influential factors, such as diet, built environment, and transportation, were not included. Additionally, repeated measures of eGFR and some potential covariates were absent. The exclusion of these factors may have constrained the precision of our results.

Finally, this study exclusively examined the SDH indicators influencing CKD within the overall population. It is plausible that variations in the composition of SDH indicators exist across different subgroups, necessitating further investigation into the underlying causes of SDH and CKD within these subpopulations.

## 5 Conclusion

In summary, the study shows that financial support, pensions, education, marital status, and medical insurance significantly affect CKD risk. Improving income, pension coverage, education, marital stability, and insurance could reduce this risk. The count of adverse SDH indicators effectively gauges an individual's SDH level and correlates with CKD risk. More indicators mean higher CKD risk, with four or more indicating a high risk. Thus, adverse SDH indicators can predict CKD.

## Data Availability

Publicly available datasets were analyzed in this study. This data can be found here: https://charls.charlsdata.com/.
